# Intraphagolysosomal conditions predispose to *Staphylococcus epidermidis* small colony variants persistence in macrophages

**DOI:** 10.1371/journal.pone.0207312

**Published:** 2018-11-09

**Authors:** Agnieszka Magryś, Kamil Deryło, Agnieszka Bogut, Alina Olender, Marek Tchórzewski

**Affiliations:** 1 Chair and Department of Medical Microbiology, Medical University of Lublin, Lublin, Poland; 2 Department of Molecular Biology, Maria Curie-Skłodowska University, Lublin, Poland; Universitatsklinikum Hamburg-Eppendorf, GERMANY

## Abstract

*Staphylococcus epidermidis* small colony variants can survive inside macrophages and their survival has been proposed as a pivotal process in the pathogenesis of biomaterial associated infections. In the present study the intracellular location of clinical isolates of SCV and parental wild type strains inside macrophages was determined. Furthermore, the effect of IFN-γ and rapamycin on the level of SCV/WT as well as lysosomes colocalisation and iNOS induction in THP-activated macrophages in response to WT and SCV strains of *Staphylococcus epidermidis* were examined. It was demonstrated that SCV strain of *S*. *epidermidis* can survive and persist inside macrophages and its intracellular survival is supported by the induction of phagosomal acidification. The ability to reduce the high proportion of LysoTracker positive SCV containing phagosomes was exclusively found when IFN-γ was used. The findings suggest that IFN-γ mediates SCV killing via two distinct mechanisms, phagosome alkalisation and an increased iNOS synthesis, so the cytokine may control *S*. *epidermidis* WT and SCV infection in macrophages. *Staphylococcus epidermidis* SCV is a less potent stimulus of iNOS than the WT strain and the feature may help SCV to persist in hostile environment of macrophages. Rapamycin treatment did not influence the iNOS synthesis but reduced the percentage of both bacterial strains within acidic organelles. However, the percentage of SCV within LysoTracker positive organelles, even though reduced comparing to non-primed cells, was higher than in the WT strain indicating that *Staphylococcus epidermidis* possesses unique metabolic features allowing SCV to survive within macrophages.

## Introduction

It may come as a surprise that a relatively avirulent skin commensal, *Staphylococcus epidermidis* is commonly involved in persistent and relapsing infections in patients receiving medical devices [1, 2]. It was reported that *Staphylococcus epidermidis* can survive inside macrophages and its survival has been proposed as a pivotal process in the pathogenesis of these infections [3].

The most deeply studied bacterial form specifically adapted for an intracellular lifestyle is the small colony variant (SCV) phenotype. The SCVs show different characteristics when compared with the parental wild type (WT), including reduced metabolic activity and a small colony size. SCVs are frequently defective in the electron transport chain components–cytochrome or menaquinone due to mutations in hemin and menadione biosynthesis genes [4, 5, 6]. Loss of electron transport reduces, besides ATP production, also transmembrane potential, that may protect bacteria from the effect of many bactericidal products. *Staphylococcus epidermidis* SCV when taken up by the host’s phagocytes is exposed to the intracellular defence mechanisms such as cationic antimicrobial proteins. The reduced susceptibility to these defence systems then provides the SCVs with a selective advantage over the wild type bacteria contributing to their survival [7]. Other auxotrophic SCVs are occasionally also isolated (for example CO_2_ auxotrophs), and SCVs for which the metabolic defects cannot be defined have also been found clinically [8].

Adaptation to intracellular lifestyle allows SCVs for evasion of the host humoral immune responses and makes them less accessible to antibiotics [9, 10]. However, within an infected cell, a pathogen is further challenged by intracellular defence mechanisms. Activated macrophages provide the least hospitable environment [10]. In phagocytes, nitric oxide (NO), produced by inducible NO synthase (iNOS) is a central mechanism of the host defence against a number of intracellular pathogens [11, 12, 13]. The expression of the enzyme can be induced by a variety of immunological and inflammatory stimuli [11]. iNOS is upregulated following stimulation of cells with proinflammatory cytokines such as IFN-γ, and bacterial cell wall components. When iNOS is upregulated, abundant NO is formed for long periods at a bactericidal level, reacting with key proteins and lipids in microbes. Since SCVs are able to survive and persist in the cells equipped to destroy them, resistance to these forces might be a prerequisite [6].

In the present study the intracellular location of clinical isolates of SCV and parental WT strains inside macrophages was determined. Furthermore, the effect of IFN-γ and rapamycin on the level of SCV/WT as well as lysosomes colocalisation and iNOS induction in THP-activated macrophages in response to WT and SCV strains of *Staphylococcus epidermidis* were examined.

## Materials and methods

### Bacterial strains and culture conditions

*Staphylococcus epidermidis* SCV, hemin defective, and a WT strain have been isolated in parallel from a single patient with the prosthetic hip joint infection and previously described in details [14]. Relatedness between the SCV and the normal-morphology isolate was assessed with the use of a commercially available DNA fingerprinting assay based on rep-PCR technology. For infection experiments, *S*. *epidermidis* strains were grown in 5 ml of tryptic soy broth (TSB; Oxoid) to the mid log phase (OD_600_ = 0.6) at 37°C under constant rotation. The cells were then harvested by centrifugation (5,000 x g for 10 min.) and washed twice in 5 ml of Dulbecco's Balanced Salt Solution (DPBS, PanBiotech). Bacterial chains and aggregates were broken by mild sonication for 3 x 10 s at 30W (Bransonic ultrasonic cleaner; G. Heinemann) at a temperature of 20°C. Samples were then centrifuged (5,000 x g for 10 min.) and the pellets were resuspended in RPMI 1650 medium (SIGMA Aldrich) without antibiotics or antimycotics. The accuracy of preparation of bacterial samples for internalization assay was routinely verified by plating dilutions on agar plates and counting colonies to determine colony forming units (CFU) per ml.

The stability of the SCV phenotype was assessed by checking colony phenotype and haemin auxotrophy over six successive subcultures. Revertants exhibiting all characteristics of a normal *S*. *epidermidis* phenotype, were excluded from the study.

To determine bacterial phenotype within infected macrophages, the infected cells were lysed after 2 h and 24 h p.i., thereby releasing intracellular bacteria, and placed on Triptic Soy Agar (TSA) plates. Next, the percentage of SCV and WT populations evolving in the presence of macrophages were defined. When SCV strain was inoculated with THP-1 derived macrophages over the course of 2 h, no phenotypic switch to normal growth was observed. After 24 h of inoculation, 7% of SCV bacteria reverted to normal phenotype in non-primed cells. Similar results were observed after rapamycin pretreatment (7%). SCV had a 12% reversion frequency to normal growth in IFN-γ primed macrophages (data not shown).

### Preparation of FITC-labelled bacteria

Overnight cultures of bacteria prepared as above, were suspended into phosphate-buffered saline (PBS) and stained with 100 μg/ml FITC (SIGMA Aldrich) in DMSO. Bacteria were incubated at room temperature in the dark with end-over-end rotation for 30 min. After incubation, bacterial cells were washed three times in PBS to remove unbound dye and resuspended in 1 mL of PBS.

### Cell culture and differentiation

THP-1 (ATCC TIB202, LGC Standards), a human monocytic cell line, was maintained in a continuous culture in RPMI 1640 medium (SIGMA Aldrich) containing 10% heat-inactivated fetal bovine serum (FBS; SIGMA Aldrich). THP-1 cells were pretreated with 10 ng/ml of phorbol 12-myristate 13-acetate (PMA; SIGMA Aldrich) for 24 h in 5% CO2 at 37°C to induce maturation of the monocytes into macrophage-like adherent cells. This procedure was followed by 2 washes (RPMI 1640) and the addition of complete media over 48 h post differentiation.

### Confocal microscopy of living cells

THP-1 activated macrophages were seeded in 35 mm cell imaging dishes with a glass bottom (Greinder BioOne) at a concentration of 3 x 10^5^ cells and incubated with rapamycin (50 nM) at the time when bacteria were added to cell culture or with recombinant IFN-γ (2000 U/ml) overnight. Macrophages were exposed to FITC-labelled *S*. *epidermidis* WT and SCV strains at MOI 25. Simultaneously, the non-stimulated cells were incubated with the same bacterial strains. After 2h of contact, the reaction was stopped by chilling on ice. Extracellular bacteria were killed by adding lysostaphin (50 μg/ml) to the culture medium. The cells were analysed for intracellular localisation of *S*. *epidermidis* SCV and WT strains after 2 h and 24 h.

To stain lysosomes in live macrophages, cells were treated with 250 nM LysoTracker Red (ThermoFisher Scientific). The host cell nuclei were stained with Hoechst 33342 (2.5 μg/ml) (Invitrogen).

Confocal live-cell imaging was conducted on LSM780 Zeiss confocal system coupled to AxioObserver Z.1 inverted microscope equipped with a Plan-Apochromat 63x/1.40 Oil DIC M27 objective and an environmental chamber to control air temperature, CO_2_ concentration, and humidity. Three-channel imaging was performed using as an excitation source 405 nm laser light for Hoechst 33342 (blue channel), 488 nm laser light for FITC (green channel), 561 nm laser light for LysoTracker Red (red channel) and PMT detectors working at 410–450 nm range, 500–550 nm range and 580–680 nm range, respectively. Simultaneously images of cells in transmission light mode were collected according to already established experimental set-up [15].

Image analysis was performed using Fiji (ImageJ, v1.52a, 64 bit Windows) software with JACoP integrated plugin. Experiments were performed in triplicate in 3 biological experiments.

### Nitric oxide synthase 2 assay

The inducible nitric oxide synthase (iNOS) level was determined in supernatants of THP-1 activated macrophages. The cells were pre incubated with rapamycin (50 nM) at the time when bacteria were added to the cell culture or with recombinant IFN-γ (2000 U/ml) overnight. Macrophages were exposed to *S*. *epidermidis* WT and SCV strains at MOI 25. Simultaneously, the non-stimulated cells were incubated with the same bacterial strains. The NOS2 level was measured after 0, 2, 24 h post infection with commercially available ELISA (Cloud Clone Corp.) according to the manufacturer’s recommendation.

### Statistics

Statistical analyses were performed using 2-tailed unpaired t-test (2 groups) or one-way ANOVA followed by Tukey’s multiple comparisons post test. P<0.05 was considered statistically significant. All data are described as mean ± SD in the text.

## Results

### *Staphylococcus epidermidis* SCV persists in lysosomes

**Fig 1:**
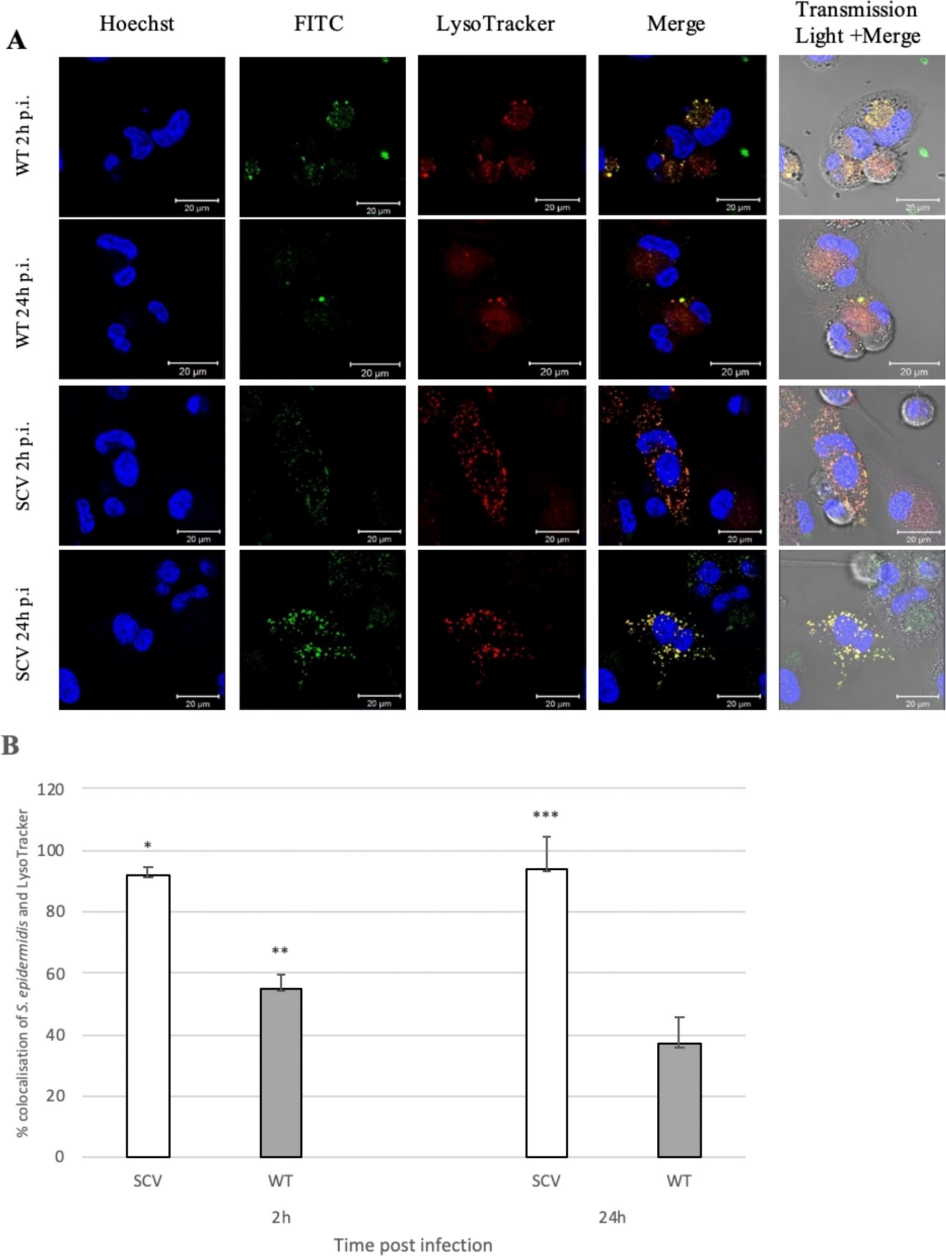
Intracellular colocalisation of *Staphylococcus epidermidis* WT and SCV strains within macrophages. THP-1 activated cells were infected with *S*. *epidermidis* WT and SCV strains, respectively at MOI 25, and extracellular bacteria were killed by addition of lysostaphin (50 μg/mL). The number of intracellular persisting bacteria were determined at indicated time points (2 h, 24 h). **(A)** The intracellular localisation of persisting bacteria (FITC; green) was analysed by confocal microscopy. Lysosomes were visualized with LysoTracker Red (red) and nuclei were stained with Hoechst (blue). Scale bar 20 μm. **(B)** Quantitative analysis of colocalisation of LysoTracker with *S*. *epidermidis* WT and SCV strains in macrophages at 2 h and 24 h post infection. Colocalisation was confirmed using Fiji (ImageJ, v1.52a, 64 bit Windows) software with JACoP integrated plugin. Values indicate the percentages of phagosomes containing *S*. *epidermidis* SCV (white bars) and WT (grey bars) colocalised with LysoTracker Red. Data are mean values (± standard deviation of the mean) performed in triplicate. *p<0.05 SCV 2 h vs. WT 2 h; **p<0.05 WT 2 h vs. WT 24 h; ***p<0.005 SCV 24 h vs. WT 24 h.

The purpose of this study was to identify the location of *S*. *epidermidis* SCV that are able to persist inside phagocytic cells. Therefore, we analysed the colocalisation of FITC-labelled bacteria with LysoTracker Red, an acidotrophic acid dye that serves as an indicator of phagosome pH. LysoTracker Red freely permeates cell membranes and remains trapped in acidic compartments upon protonation. In THP-1 derived macrophages, bacteria were mostly included in a LysoTracker positive compartments. As shown in Fig 1A and 1B, 55% of organelles containing WT bacteria colocalised with LysoTracker after 2 h post infection (p.i.). This colocalisation level significantly decreased to 37% over the period of 24 h (p<0.05). In contrast, a significantly higher percentage of phagosomes containing SCV strains of *S*. *epidermidis* (92%) colocalised with LysoTracker at 2 h post infection and remained constant till last time analysed (Fig 1A and 1B).

These results indicate that while the wild type parental strain of *S*. *epidermidis* is degraded in acidic phagocytes, SCV strain can survive in acidic compartments for at least 24 h.

According to our previous observation, the CFU/ml of WT bacteria decreased in large amounts within the first 24 h post-phagocytosis, whereas the number of alive SCV was barely reduced during the study time [16].

### Priming macrophages with IFN-γ helps macrophages eradicate intracellular *Staphylococcus epidermidis* WT and SCV

**Fig 2:**
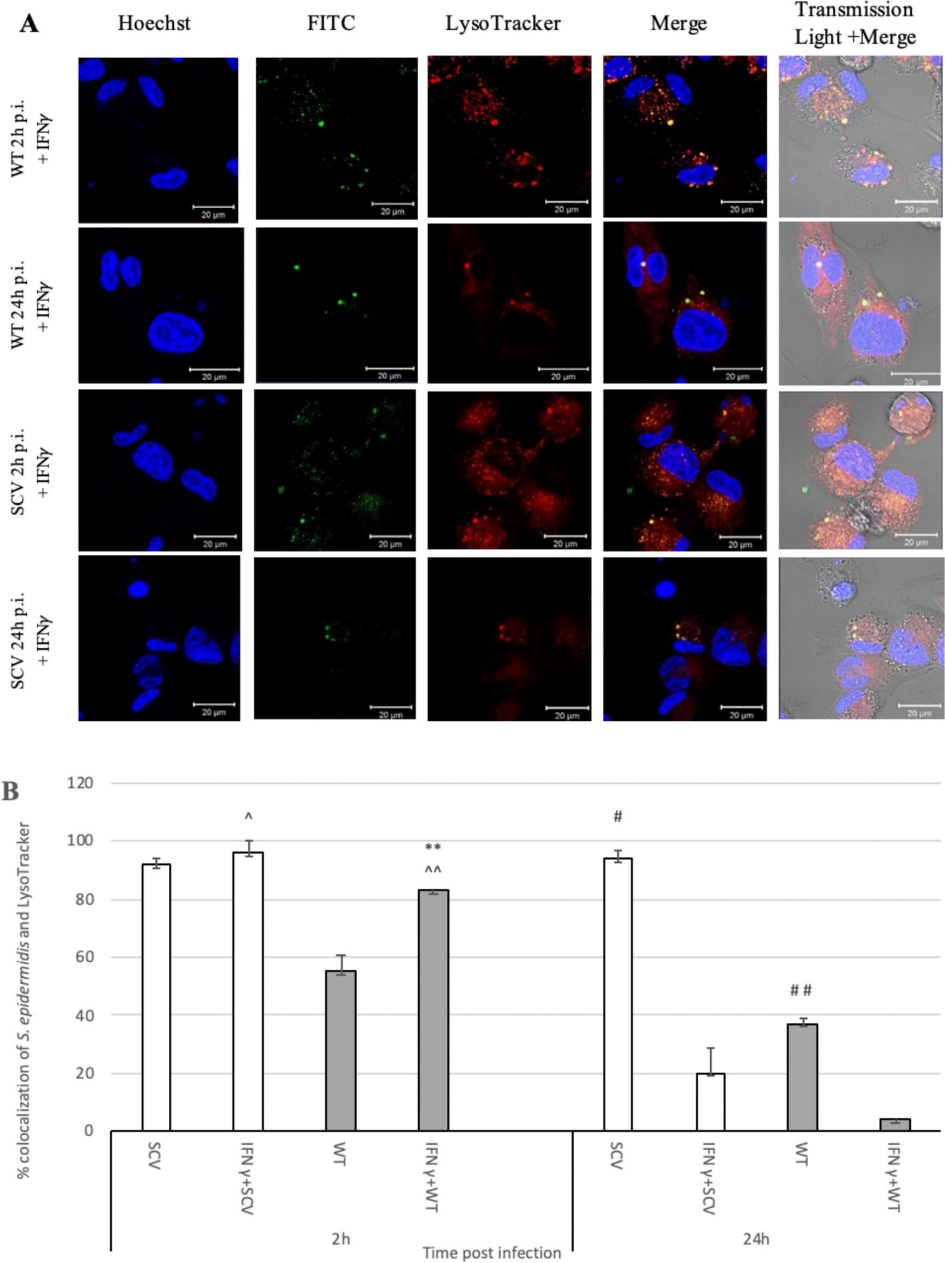
Intracellular colocalisation of *Staphylococcus epidermidis* WT and SCV strains within macrophages. THP-1 activated cells were primed with IFN-γ for 24 h and next, infected with *S*. *epidermidis* WT and SCV strains, respectively at MOI 25. Extracellular bacteria were killed by addition of lysostaphin (50 μg/mL). **(A)** The intracellular localisation of persisting bacteria (FITC; green) was analysed by confocal microscopy. Lysosomes were visualized with LysoTracker Red (red) and nuclei were stained with Hoechst (blue). Scale bar 20 μm. **(B)** Quantitative analysis of colocalisation of LysoTracker with *S*. *epidermidis* WT and SCV strains in IFN-γ treated macrophages at 2 h and 24 h post infection. Colocalisation was confirmed using Fiji (ImageJ, v1.52a, 64 bit Windows) software with JACoP integrated plugin. Values indicate the percentages of phagosomes containing *S*. *epidermidis* SCV (white bars) and WT (grey bars) colocalised with LysoTracker Red. Data are mean values (± standard deviation of the mean) performed in triplicate. **p<0.05 WT 2 h vs. IFN+WT 2 h; # p<0.05 SCV 24 h vs. IFN+SCV 24 h; # #p<0.05 WT 24 h vs. IFN+WT 24 h; ^p<0.05 IFN+SCV 2 h vs. IFN+SCV 24 h; ^^ IFN+WT 2 h vs. IFN+WT 24 h.

Considering that IFN-γ plays a pivotal role in immune response against intracellular pathogens, here it was tested whether stimulation of macrophages with the cytokine affects the host cell-pathogen interaction towards more efficient colocalisation and killing abilities. When THP-1 derived macrophages were stimulated with IFN-γ, a high number of LysoTracker positive, acidic organelles was observed 2 h p.i. and that effect was decreased over 24 h. FITC-labelled SCV and WT bacteria colocalised with LysoTracker and the number of internalised bacteria, both SCV and WT colocalised with LysoTracker positive organelles significantly decreased by 24 h post infection (p<0.05) (Fig 2A and 2B). These observations suggest that IFN-γ affects the macrophage-*S*. *epidermidis* interaction in the way resulting in efficient colocalisation with LysoTracker positive organelles 2 h p.i. significant decrease in the number of colocalised bacteria after 24h p.i., irrelevant of the bacterial phenotype (p<0.05).

### Rapamycin decreases *Staphylococcus epidermidis* WT and SCV phagosome colocalisation

Rapamycin as a specific inhibitor of mammalian target of rapamycin (mTOR), has immune suppressive effect and increases autophagy. For this reason, blocking mTOR may contribute to the control of intracellular pathogens [17, 18].

**Fig 3:**
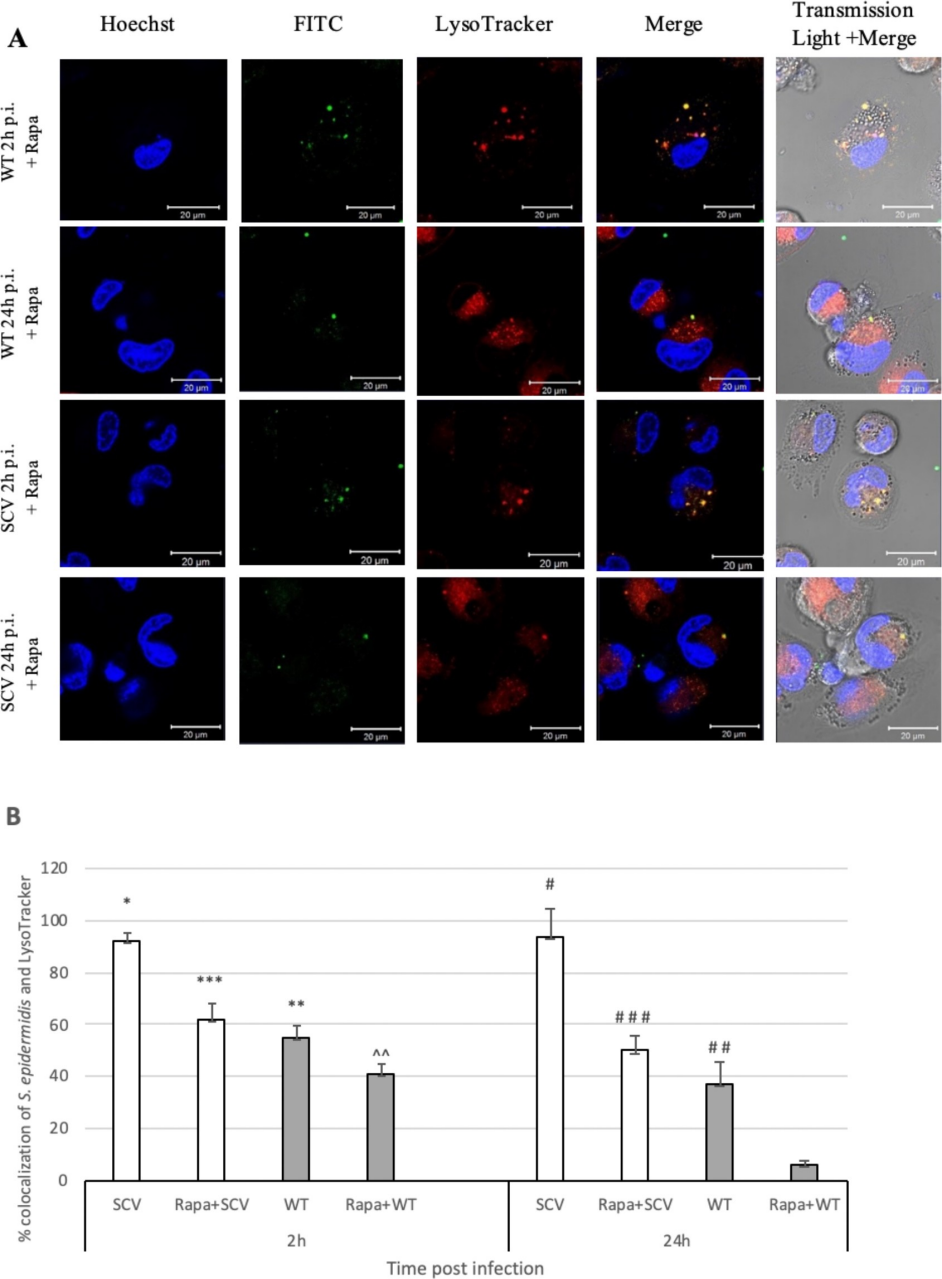
Intracellular colocalisation of *Staphylococcus epidermidis* WT and SCV strains within macrophages. THP-1 activated cells were pre incubated with rapamycin (50 nM) at the time when bacteria were added to cell culture and next, infected with *S*. *epidermidis* WT and SCV strains, respectively at MOI 25. Extracellular bacteria were killed by addition of lysostaphin (50 μg/mL). **(A)** The intracellular localisation of persisting bacteria (FITC; green) was analysed by confocal microscopy. Lysosomes were visualized with LysoTracker Red (red) and nuclei were stained with Hoechst (blue). Scale bar 20 μm. **(B)** Quantitative analysis of colocalisation of LysoTracker with *S*. *epidermidis* WT and SCV strains in rapamycin treated macrophages at 2 h and 24 h post infection. Colocalisation was confirmed using Fiji (ImageJ, v1.52a, 64 bit Windows) software with JACoP integrated plugin. Values indicate the percentages of phagosomes containing *S*. *epidermidis* SCV (white bars) and WT (grey bars) colocalised with LysoTracker Red. Data are mean values (±standard deviation of the mean) performed in triplicate. *p<0.05 SCV 2 h vs. Rapa + SCV 2 h; **p<0.05 WT 2 h vs. Rapa + WT 2 h; ***p<0.05 Rapa+SCV 2 h vs. Rapa+WT 2 h; # SCV 24 h vs. Rapa+WT 24 h; # #p<0.05 WT 24 h vs. Rapa+WT 24 h; # # #p<0.05 Rapa+SCV 24 h vs. Rapa+WT 24 h; ^^ Rapa+WT 2 h vs. Rapa+WT 24 h.

In the previous paper [19], incubation of *S*. *epidermidis* SCV and WT strains with rapamycin pretreated macrophages resulted in a significant decrease in bacterial CFU. In a recent work it was analysed whether the stimulation of autophagic pathway by rapamycin would modify the colocalisation of bacteria with LysoTracker positive organelles. When macrophages were treated with rapamycin, the proportion of SCV that colocalised with LysoTracker significantly decreased comparing to untreated cells from 92% to 62% at 2 h p.i. (p<0.05) (Fig 3A and 3B) and from 94% in untreated macrophages to 50% in rapamycin-treated cell at 24 h p.i. (p<0.05) (Fig 3A and 3B). Similarly, rapamycin treatment of macrophages infected with the WT strain decreased the level of bacterial colocalisation with LysoTracker from 55% in untreated cells to 41% (p<0.05) in rapamycin-treated cells 2 h p.i. and from 37% to 6% after 24 h p.i. (p<0.05). Of interest, rapamycin-treated macrophages showed the higher proportion of colocalised SCV with phagosomes than the WT strain at 2 and 24 h p.i. Also, the percentage of SCV colocalised with LysoTracker positive organelles did not significantly change during the study period unlike the WT strain (p<0.05).

These results indicate that, in cells incubated under conditions that favour autophagy, colocalisation of *S*. *epidermidis* within acidic organelles is reduced. Moreover, treatment of macrophages with rapamycin did not significantly affect the proportion of the SCV strain during study time.

### Decreased level of iNOS after *Staphylococcus epidermidis* SCV internalization

An effective host response to pathogens requires capable function of immune cells in locating, phagocytosis and microbial killing by releasing a variety of effector molecules, including reactive oxygen and nitrogen species [13].

**Fig 4:**
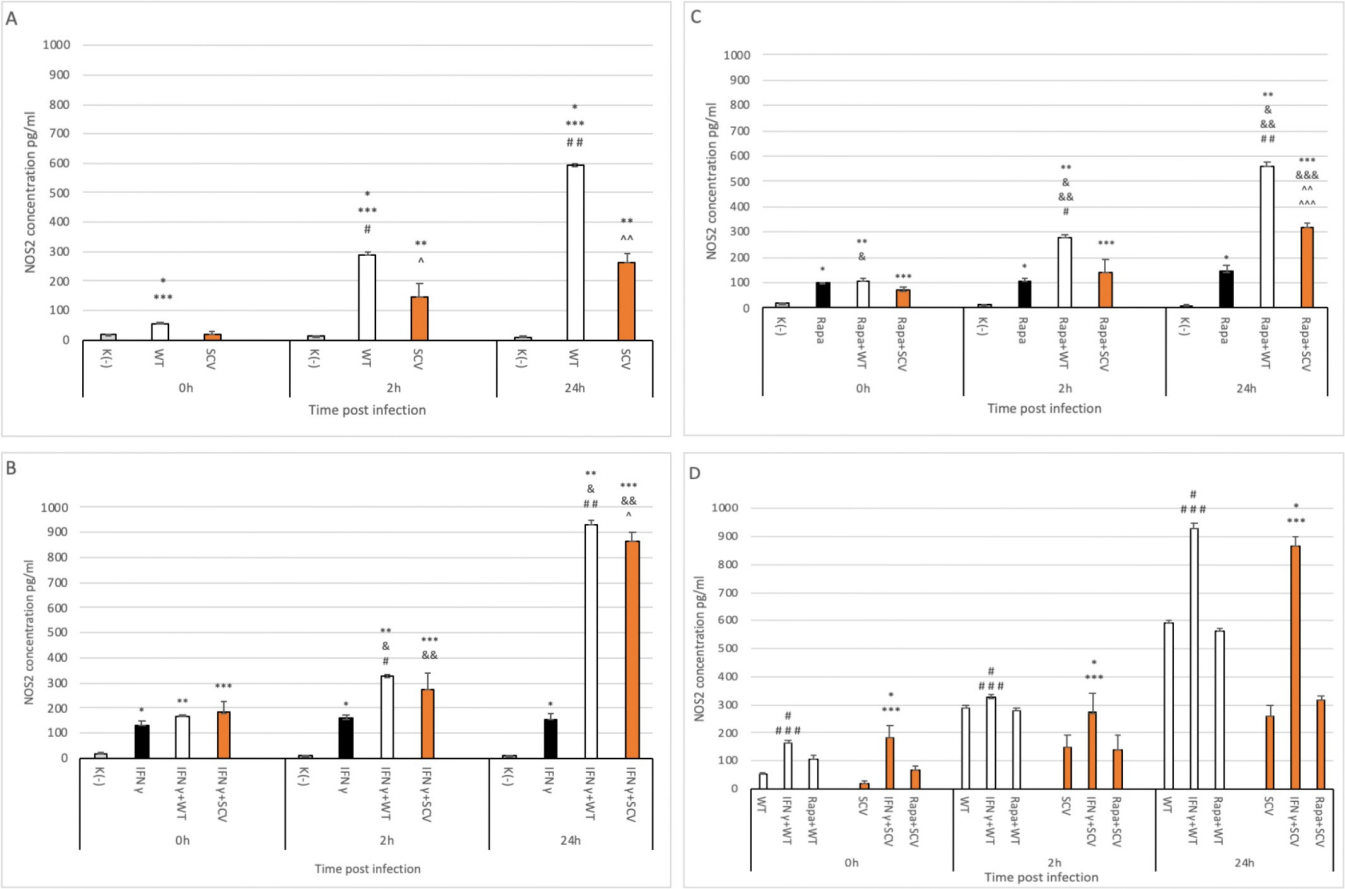
Effects of different stimuli on iNOS synthesis in THP-1 activated macrophages. **(A)** Macrophages were activated with WT and SCV strains respectively for 0 h, 2 h, 24 h. Data are expressed as means ± SD and are representative of 3 independent experiments. K indicates negative control of untreated and unstimulated cells. Where indicated *p<0.05 K vs. WT; **p<0.05 K vs. SCV; ***p<0.05 WT vs. SCV; #p<0.05 WT 0 h vs. WT 2 h; # #p<0.05 WT 2h vs. WT 24 h; ^p<0.05 SCV 0 h vs. SCV 2 h; ^^p<0.05 SCV 2 h vs. SCV 24 h. **(B)** IFN-γ primed cells were infected with bacterial strains for indicated time points post infection. Data are expressed as means ± SD and are representative of 3 independent experiments. K indicates negative control of untreated and unstimulated cells. IFN indicates macrophages pre-treated with IFN-γ. Where indicated *, **, ***p<0.05 all relative to control (K); &p<0.05 IFN + WT vs. IFN; &&p<0.05 IFN + SCV vs. IFN relative to the same time p.i.; #p<0.05 IFN + WT 0h vs. IFN + WT 2h; # #p<0.005 IFN + WT 2 h vs. IFN + WT 24 h; ^p<0.05 IFN + SCV 2 h vs. IFN + SCV 24h. **(C)** cells were treated with WT or SCV in presence of rapamycin for 0 h, 2 h, 24 h post infection. iNOS synthesis was assessed by ELISA in the incubation medium. Data are expressed as means ± SD and are representative of 3 independent experiments. Data are expressed as means ± SD and are representative of 3 independent experiments. K indicates negative control of untreated and unstimulated cells. Rapa indicates macrophages treated with rapamycin. Where indicated *, **, ***p<0.05 all relative to control (K); &p<0.05 Rapa + WT vs. Rapa + SCV; &&p<0.05 Rapa + WT vs. Rapa; &&&p<0.05 Rapa + SCV vs. Rapa relative to the same time p.i.; #p<0.05 Rapa + WT 0h vs. Rapa + WT 2h; # #p<0.005 Rapa + WT 2 h vs. Rapa + WT 24 h; ^^p<0.05 Rapa + SCV 0h vs. Rapa + SCV 24h; ^^^p<0.05 Rapa + SCV 2 h vs. Rapa + SCV 24h. **(D)** Effect of priming with IFN-γ and rapamycin on iNOS concentration in relation to *S*. *epidermidis* WT and SCV. Data are expressed as means ± SD and are representative of 3 independent experiments. Where indicated #p<0.05 WT vs. IFN + WT; # # #p<0.05 IFN + WT vs. Rapa + WT; *p<0.05 SCV vs. IFN + SCV; ***p<0.05 IFN + SCV vs. Rapa + SCV.

The next stage was to detect iNOS, an enzyme that synthesises NO, in bacteria activated macrophages. Under resting conditions, iNOS was undetectable in THP-activated macrophages. Exposure of cells to WT and SCV strains resulted in a significant increase in the concentration of iNOS after 0 h (WT strain), 2 h (WT and SCV strains) and 24 h (WT and SCV strains) when compared to control, uninfected cells (p<0.05) (Fig 4A). Moreover, the concentration of iNOS after bacterial infection increased significantly during study time irrespective of *S*. *epidermidis* strain.

Macrophages can be activated by IFN-γ *in vitro* to express enhanced antimicrobial activity [20]. Therefore, the effect of the cytokine on the concentration of iNOS after infection with *S*. *epidermidis* WT and SCV was checked. As seen in Fig 4B, IFN-γ primed macrophages expressed higher iNOS than non-stimulated cells (p<0.05).

Significant amounts of iNOS were detected after IFN-γ pretreatment of THP-activated cells and were significantly increased by WT and SCV strains during study time (p<0.05) what may facilitate in killing mechanisms used by the host to eradicate attacking bacteria. Next, the effect of rapamycin in WT and SCV infected macrophages on iNOS synthesis was investigated. In rapamycin pretreated macrophages the level of iNOS increased after 0 h, 2 h and 24 h post internalisation of WT strain when compared to control untreated cells and cells treated with rapamycin only (p<0.05; Fig 4C). Inhibition of mTOR by rapamycin displayed only minor effects on SCV infected macrophages after 0 and 2 h post internalisation, but the level of the enzyme significantly increased after 24 h post infection with the SCV strain (p<0.05; Fig 4C). Of note, the concentration of the enzyme was comparable to the level when bacteria alone were incubated with the cells, regardless of bacterial strain (Fig 4D).

## Discussion

Phagocytosis is among the most important mechanisms used for elimination of invading pathogens, and belongs to immunological cellular response representing the first line of defence. Professional phagocytes, such as macrophages play an important role in eliminating pathogens by exposing them to intracellular defence mechanisms [3, 10]. To survive and replicate inside macrophages *S*. *epidermidis* Small Colony Variants, regularly isolated from recurrent and persistent infections, have developed strategies to subvert the host’s defence mechanisms [5, 9]. Taking into account the obvious intracellular persistence of *S*. *epidermidis* haemin auxotrophic SCV strain its intracellular localisation and fate was investigated as compared to the normal parental phenotype. In the model system, using THP-activated macrophages essentially all of the intracellular *S*. *epidermidis* WT and SCV strains accumulated in acidic compartments within 2 h p.i. LysoTracker, a dye staining specifically with acidic organelles, was previously shown to occupy compartments that were consistent with features of late endosomes and lysosomes [5, 21]. In our experiment, in the course of the study, phagosomes containing WT bacteria showed a dramatically decreased level of colocalisation with LysoTracker within 24 h p.i. In contrast, the number of SCV remained stable during the study time. These data indicate that low pH in phagosomes exert an effective antibacterial activity towards the WT strain of *S*. *epidermidis*, whereas, the SCV of *S*. *epidermidis* can persist within phagocytic milieu of macrophages which may contribute to the bacterial ability to persist and evade host defences. At present, it is not clearly known what enables SCVs to survive inside such a hostile environment. It has been proposed that SCV properties, such as decreased transmembrane potential or altered cell wall composition, may protect them from bactericidal products of macrophages [5]. Moreover, it was shown that SCV formation is triggered by harsh conditions, such as antibiotic pressure or exposure to acidic conditions [4, 22].

One important bactericidal mechanism against intracellular pathogens is the release of nitric oxide produced by iNOS in macrophages [23, 24]. Results suggest that there are major differences in synthesis of iNOS in response to WT and SCV strains of *S*. *epidermidis* and reduced iNOS production after SCV infection may in part explain bacterial persistence. SCV strains are killed by the NO less efficiently probably by having low capacity to stimulate iNOS synthesis and being more resistant to cellular microbicides.

In the present study it is also investigated whether bactericidal effects inside phagocyte may be affected by IFN-γ and rapamycin stimulation.

The obtained data show that priming with IFN-γ for 24 h has several important effects on the interaction between *S*. *epidermidis* WT and SCV and THP-activated macrophages. First, the stimulation of macrophages with the cytokine resulted in a high proportion of colocalisation of the WT and SCV strains with LysoTracker positive organelles after 2 h p.i. with their significant reduction after 24 h p.i. The high proportion of lysosome-associated WT and SCV bacteria in the present study may be explained by the fact suggested by Gordon et al. implying that IFN-γ increases the process of bacterial adherence and internalisation by an increase in the number or density of surface receptors on the cells [25]. Despite a high number of colocalised WT and SCV strains in acidic organelles in IFN-γ primed cells, there was a significant reduction in the percentage of colocalised WT and SCV bacteria with LysoTracker positive organelles over the 24 h period of the study. Moreover, it was found that 12% of SCV bacteria reverted to normal growth within infected macrophages during study period. Reduction in LysoTracker positive organelles density as a consequence of IFN-γ priming, 24 h post infection was also observed. Leimer found that rising pH within phagolysosomes reverted *S*. *aureus* SCV strain to normal phenotype making it more vulnerable to intracellular antibiotics [22]. Phagolyzosomal alkalization is also an effective strategy for eradicating other obligate intracellular pathogens, *Coxiella burnetti* [26]. When IFN-γ was added before cell infection, it was able to stimulate *C*. *burnetii* killing but it also induced vacuolar alkalization [26]. Considering that phagosomal acidification is essential for the intracellular survival of SCV and that those can easily persist at low pH, the results suggest that priming macrophages with proinflammatory cytokine stimulates efficient anti-bacterial strategies to mediate intracellular killing of both *S*. *epidermidis* phenotypes. IFN-γ as an effective inducer of antimicrobial strategies of macrophages prevented intracellular persistence showing significant reduction in bacterial colocalisation 24 h p.i.

Secondly, as a consequence of pre-exposure of macrophages to IFN-γ, an increase in iNOS synthesis was observed 24 h p.i. Priming with IFN-γ gives the uninfected macrophages powerful advantages against future bacterial infection [27]. It has been demonstrated that iNOS expression was weakly upregulated by SCV *per se*. However, upon induction with IFN-γ, a potent inflammatory stimulus, SCV augmented synthesis of iNOS at a level similar to WT. These results confirm that priming enables macrophages to produce proinflammatory response against *S*. *epidermidis*, irrelevant of its phenotype [27]. This, in part, may be related to the phenotype switch, as 12% of studied *S*. *epidermidis* SCV reverted to fast growth in IFN-γ stimulated macrophages after 24h of inoculation. The mechanisms by which IFN-γ limits the amount of intracellular bacteria in macrophages is not limited to iNOS expression with subsequent NO production, and multiple antibacterial mechanisms combine to mediate pathogens killing. Beekhuizen found that IFN-γ inhibits intracellular replication of *S*. *aureus* by limiting availability of essential nutrients such as iron and tryptophan [28]. Killing of intracellular bacteria by the cytokine activated macrophages is also aided by anti-microbial peptides and is linked to autophagy [23].

Autophagy, as a fundamental process in eukaryotic cells normally maintains cellular homeostasis by degrading damaged or unnecessary cytosolic components and recycling the resulting metabolites and maintaining ATP production [17, 29, 30]. Apart from its function maintaining cellular homeostasis, cells use autophagy as an anti-infective machinery for the clearance of intracellular bacteria [17, 29, 30, 31]. Autophagic response is triggered by many intracellular pathogens, including *Shigella*, *Salmonella*, *Listeria* or *Mycobacterium* that at least in part inhibits their intracellular replication. However, the same pathogens can actively suppress autophagic process and survive within the host cells [17, 31].

Autophagy is regulated by multiple pathways and a master regulatory kinase, mTOR plays a crucial role in this process. mTOR impacts several cellular functions, stimulating synthesis of proteins, lipids and nucleotides and blocking catabolic processes such as autophagy [18]. On the other hand, its activity is suppressed when cells are starved for nutrients, lack cellular energy or amino acids which results in an increase of autophagy and inhibition of protein translation [17, 29]. It is confirmed that mTOR activity is also suppressed during infection of macrophages by pathogenic bacteria, which suggests that the process may be beneficial for the host cell [17, 29]. Thus, this finding prompted the authors to study the role of rapamycin-mediated inhibition of mTOR in the fate of *S*. *epidermidis* WT and SCV strains in macrophages. The results revealed that pharmacological inhibition of mTOR by rapamycin resulted in the overall decrease in *S*. *epidermidis* WT and SCV colocalisation within phagosomes comparing to non-primed cells. However, rapamycin treatment did not significantly influence the percentage of SCV containing phagosomes during the study time, whereas the proportion of LysoTracker positive WT containing organelles was drastically decreased 24 h p.i. These data indicate that the proportion of bacteria in phagosomes can be modulated using rapamycin, a known inhibitor of mTOR. Although a decrease in *S*. *epidermidis* SCV and WT colocalisation with LysoTracker positive phagosomes was observed in rapamycin-treated cells as soon as 2 h post infection, the colocalisation of SCV with acidic organelles was significantly higher when compared to its parental WT strain. This may indicate that WT strain of *S*. *epidermidis* is more susceptible to autophagic killing than SCV strain so SCV seems to be able to, in part, subvert the autophagy pathway induced by rapamycin. But additional experiments are required to completely understand the role of autophagy in WT and SCV infections.

Regardless of the *S*. *epidermidis* strain infecting macrophages, rapamycin did not modify the iNOS level and the amount of the enzyme was comparable to the level when bacteria alone were incubated with the cells. These findings clearly indicate that blocking of mTOR signaling pathway is involved in regulation of the iNOS synthesis and that the effect observed can be regarded as anti-inflammatory. In accordance with the presented report, it has been shown that rapamycin down regulates LPS-induced iNOS protein expression and subsequent NO production in mouse macrophages [32, 33]. It is suggested that rapamycin reduces the level of iNOS by a dual mechanism involving mTOR signaling and by promoting its degradation through 20S proteosomal activation [33].

## Conclusions

In conclusion, it was demonstrated that haemin auxotrophic SCV strain of *S*. *epidermidis* can persist inside macrophages and its intracellular persistence is supported by the induction of phagosomal acidification. The ability to reduce the high proportion of LysoTracker positive SCV containing phagosomes was exclusively found when IFN-γ was used. The findings suggest that IFN-γ mediates SCV killing via two distinct mechanisms, phagosome alkalisation and an increased iNOS synthesis, so the cytokine may control *S*. *epidermidis* WT and SCV infection in macrophages. *Staphylococcus epidermidis* SCV is a less potent stimulus of iNOS than the WT strain and the feature may help SCV to persist in hostile environment of macrophages. Rapamycin treatment did not influence the iNOS synthesis but reduced the percentage of both bacterial strains within acidic organelles. However, the percentage of SCV within LysoTracker positive organelles, even though reduced comparing to non-primed cells, was higher than in the WT strain indicating that *Staphylococcus epidermidis* possesses unique metabolic features allowing haemin auxotrophic SCV to survive within macrophages.

## References

[1] Khalil H, Willians RJ, Stenbeck G, Henderson B, Meghji S, Nair SN. Invasion of bone cells by *Staphylococcus epidermidis*. Microbes Infect. 2007; 9(4): 460–465. 10.1016/j.micinf.2007.01.002 17331787

[2] Bresco MS, Harris LG, Thompson K, Stanic B, Morgensten M, O’Mahony L, et al Pathogenic mechanisms and host interactions in *Staphylococcus epidermidis* device-related infection. Front Microbiol. 2017; 8: 1401 10.3389/fmicb.2017.01401 28824556 PMC5539136

[3] Lacoma A, Cano V, Moranta D, Regueiro V, Dominguez-Villanueva D, Laabei M, et al Investigating intracellular persistence of *Staphylococcus aureus* within a murine alveolar macrophage cell line. Virulence. 2017; 8: 1761–1775. 10.1080/21505594.2017.1361089 28762868 PMC5810471

[4] Proctor RA, Kriegeskorte A, Kahl BC, Becker K, Lӧffler B, Peters G. *Staphylococcus aureus* Small Colony Variants (SCVs): a road map for the metabolic pathways involved in persistent infections.Front Cell Infect Microbiol. 2014; 7 28; 4: 99 10.3389/fcimb.2014.00099 25120957 PMC4112797

[5] Schrӧder A, Kland R, Peschel A, von Eiff Ch, Aepfelbacher M. Live cell imaging of phagosome maturation in *Staphylococcus aureus* infected human endothelial cells: small colony variants are able to survive in lysosomes. Med Microbiol Immunol. 2006; 195: 1850–194. 10.1007/s00430-006-0015-0 16596413

[6] Samuelsen O, Haukland HH, Kahl BC, von Eiff Ch, Proctor RA, Ulvatne H, et al *Staphylococcus aureus* small colony variants are resistant to the antimicrobial peptide lactoferricin B. J Antimicrob Chemother. 2005; 56: 1126–1129. 10.1093/jac/dki385 16287983

[7] Mack D, Davies AP, Harris LG, Jeeves R, Pascoe B, Knobloch JK-M, et al *Staphylococcus epidermidis* in biomaterial-associated infections In: Moriarty TF, Zaat SAJ, Busscher HJ. Biomaterials associated infections. Immunological aspects and antimicrobial strategies. Springer; 2013 pp. 25–56.

[8] Proctor RA, von Eiff Ch, Kahl BC, Becker K, McNamara P, Herrman M, et al Small colony variants: a pathogenic form of bacteria that facilitates persistent and recurrent infections. Nat Rev Microbiol. 2006; 4; 4(4): 295–305. 10.1038/nrmicro1384 16541137

[9] Thompson KM, Jefferson KK. Adaptation to stress: biofilms and small colony variants In: Crossley KB, Jefferson KK, Archer GL, Fowler VG. Staphylococci in human disease. Blackwell; 2009 pp. 109–124.

[10] Kumar Y, Valdivia RH. Leading a sheltered life: intracellular pathogens and maintenance of vacuolar compartments. Cell Host Microbe. 2009; 6 18; 5(6): 593–60. 10.1016/j.chom.2009.05.014 19527886 PMC2716004

[11] Webb JL, Harvey MW, Holden DW, Evans TJ. Macrophage nitric oxide synthase associates with cortical actin but is not required to phagosomes. Infect Immun. 2001; 69(10): 6391–6400. 10.1128/IAI.69.10.6391-6400.2001 11553583 PMC98774

[12] Sasaki S, Miura T, Nishikawa S, Yamada K, Hirasue M, Nakane A. Protective role of nitric oxide in *Staphylococcus aureus* infection in mice. Infect Immun. 1998; 66(3): 1017–1022. 9488390 10.1128/iai.66.3.1017-1022.1998 PMC108010

[13] Lowenstein ChJ, Padalko E. iNOS (NOS2) at a glance. J Cell Sci. 2004; 117: 2865–2867. 10.1242/jcs.01166 15197240

[14] Bogut A, Niedźwiadek J, Kozioł-Montewka M, Strzelec-Nowak D, Blacha J, Mazurkiewicz T, et al Characterization of *Staphylococcus epidermidis* and *Staphylococcus warneri* small-colony variants associated with prosthetic-joint infections. J Med Microbiol. 2014; 63: 176–185. 10.1099/jmm.0.066068-0 24257683

[15] Deryło K, Michalec-Wawiórka B, Krokowski D, Wawiórka L, Hatzoglou M, Tchórzewski M. The uL10 protein, a component of the ribosomal P-stalk, is released from the ribosome in nucleolar stress. Biochim Biophys Acta. 2018 1; 1865(1):34–47. 10.1016/j.bbamcr.2017.10.002 28986221

[16] Magryś A, Paluch-Oleś J, Bogut A, Kiełbus M, Plewik D, Kozioł-Montewka M. The role of programmed death ligand 1 pathway in persistent biomaterial-associated infections. J Microbiol. 2015 8;53(8):544–52. 10.1007/s12275-015-5022-7 26224457

[17] Casanova JE. Bacterial autophagy: offense and defence at the host-pathogen interface. Cell Mol Gastroenterol Hepatol. 2017; 4: 237–243. 10.1016/j.jcmgh.2017.05.002 28660242 PMC5480303

[18] Young Chul Kim, Kun-Liang Guan. mTOR: a pharmacologic target for autophagy regulation. J Clin Invest. 2015; 125(1): 25–32. 10.1172/JCI73939 25654547 PMC4382265

[19] Magryś A, Bogut A, Kiełbus M, Olender A. The role of the PI3K/mTOR signaling pathway in *Staphylococcus epidermidis* small colony variants intracellular survival. Immun Invest. 2018; 47(3): 251–263. 10.1080/08820139.2018.1423569 29336620

[20] Rijneveld AW, Lauw FN, Schultz MJ, Florquin S, te Velde AA, Spleeman P, et al The role of interferon-gamma in murine pneumococcal pneumonia. J Infect Dis. 2002; 185(1): 91–97. 10.1086/338122 11756986

[21] Via LE, Fratti RA, Mc Falone M, Pagan-Ramos E, Deretic D, Deretic V. Effects of cytokines on mycobacterial phagosome maturation. J Cell Sci. 1998; 111: 897–905. 9490634 10.1242/jcs.111.7.897

[22] Leimer N, Rachmül C, Marques MP, Bahlmann AS, Furrer A, Eichenseher F, et al Nonstable *Staphylococcus aureus* small colony variants are induced by low pH and sensitized to antimicrobial therapy by phagolyzosomal alkalization. J Infect Dis. 2016; 213: 305–313. 10.1093/infdis/jiv388 26188074

[23] Herbst S, Schaible UE, Schneider BE. Interferon gamma activated macrophages kill *Mycobacteria* by nitic oxide induced apoptosis. PLoS One. 2011; 6(5): e19105 10.1371/journal.pone.0019105 21559306 PMC3085516

[24] Jubrail J, Morris P, Bewley MA, Stoneham S, Johnston SA, Foster SJ, et al Inability to sustain intraphagolysosomal killing of *Staphylococcus aureus* predisposes to bacterial persistence in macrophages. Cell Microbiol. 2016; 18(1): 80–86. 10.1111/cmi.12485 26248337 PMC4778410

[25] Gordon MA, Jack DL, Dockrell DH, Lee ME, Read RC. Gamma Interferon enhances internalization and early nonoxidative killing of *Salmonella enterica* serovar typhimurium by human macrophages and modifies cytokine responses. Infect Immun. 2005; 73(6): 3445–3452. 10.1128/IAI.73.6.3445-3452.2005 15908373 PMC1111838

[26] Ghigo E, Capo C, Tung CH, Raoult D, Gorvel JP, Mege JL. *Coxiella burnetii* survival in THP-1 monocytes involves the impairment of phagosome maturation: IFN–gamma mediates its restoration and bacterial killing. J Immunol. 2002; 169(8): 4488–4495. 12370385 10.4049/jimmunol.169.8.4488

[27] Salim T, Sershen ChL, May EE. Investigating the role of TNF-α and IFN-γ activation on the dynamics of iNOS gene expression in LPS stimulated macrophages. PLoS One. 2016; 11(6): e0153289 10.1371/journal.pone.0153289 27276061 PMC4898755

[28] Beekhuizen H, van de Gevel JS. Gamma Interferon confers resistance to infection with *Staphylococcus aureus* in human vascular endothelial cells by cooperative proinflammatory and enhanced intrinsic antibacterial activities. Infect Immun. 2007; 75(12): 5615–5626. 10.1128/IAI.00530-07 17893127 PMC2168329

[29] Vural A, Kehrl JH. Autophagy in macrophages: impacting inflammation and bacterial infection. Scientifica (Cairo). 2014; 2014:825463 10.1155/2014/825463 24818040 PMC4000662

[30] Neumann Y, Bruns SA, Rohde M, Prajsnar TK, Foster SJ, Schmitz I. Intracellular *Staphylococcus aureus* eludes selective autophagy by activating a host cell kinase. Autophagy. 2016; 12(11): 2069–2084. 10.1080/15548627.2016.1226732 27629870 PMC5103350

[31] Cullinane M, Gong L, Li X, Adler N-L, Tra T, Wolvetang E, et al Stimulation of autophagy suppresses the intracellular survival of *Burkholderia pseudomallei* in mammalian cell lines. Autophagy. 2008; 4(6): 744–753. 18483470 10.4161/auto.6246

[32] Lisi L, Navarra P, Feinstein DL, Dello Russo C. The mTOR kinase inhibitor rapamycin decreases iNOS mRNA stability in astrocytes. J Neuroinflammation. 2011; 8(1):1 10.1186/1742-2094-8-1 21208419 PMC3025854

[33] Jin HK, Ahn SH, Yoon JW, Park JW, Lee EK, Choi WS, et al Rapamycin down-regulates inducible nitric oxide synthase by inducing proteosomal degradation. Biol Pharm Bull. 2009; 32(6): 988–992. 19483303 10.1248/bpb.32.988

